# Posterior mitral leaflet hypoplasia in adults: diagnostic value of three-dimensional transoesophageal echocardiography: a case series

**DOI:** 10.1093/ehjcr/ytag160

**Published:** 2026-04-24

**Authors:** Dylan Young, Edward Dababneh, Vivek Kulkarni, Rowena Solayar, Ian Agahari, Kuljit Singh, Maria Gabriela Matta

**Affiliations:** Department of Cardiology, Division of Specialist Medical Services, Gold Coast Hospital and Health Services, Southport, QLD 4215, Australia; Department of Medicine, Griffith University School of Medicine, Parklands Dr, Southport, QLD 4215, Australia; Department of Cardiology, Division of Specialist Medical Services, Gold Coast Hospital and Health Services, Southport, QLD 4215, Australia; Department of Medicine, Griffith University School of Medicine, Parklands Dr, Southport, QLD 4215, Australia; Department of Cardiology, Division of Specialist Medical Services, Gold Coast Hospital and Health Services, Southport, QLD 4215, Australia; Department of Cardiology, Division of Specialist Medical Services, Gold Coast Hospital and Health Services, Southport, QLD 4215, Australia; Department of Cardiology, Division of Specialist Medical Services, Gold Coast Hospital and Health Services, Southport, QLD 4215, Australia; Department of Cardiology, Division of Specialist Medical Services, Gold Coast Hospital and Health Services, Southport, QLD 4215, Australia; Department of Medicine, Griffith University School of Medicine, Parklands Dr, Southport, QLD 4215, Australia; Department of Cardiology, Division of Specialist Medical Services, Gold Coast Hospital and Health Services, Southport, QLD 4215, Australia; Department of Cardiology, The Prince Charles Hospital, Brisbane, QLD 4032, Australia

**Keywords:** Posterior mitral leaflet hypoplasia, Echocardiogram, Congenital heart disease, Three-dimensional transoesophageal echocardiography, Case report, Case series

## Abstract

**Background:**

Posterior mitral leaflet (PML) hypoplasia is a rare and frequently under-recognized condition in adults, as transthoracic echocardiography (TTE) findings may be subtle. Transoesophageal echocardiography (TOE), particularly with three-dimensional (3D) imaging, is invaluable in confirming leaflet morphology and identifying associated anomalies.

**Case summary:**

We describe three adult patients with PML hypoplasia confirmed by 3D TOE. Common features included rudimentary posterior leaflet, elongated anterior leaflet, and frequent association with congenital anomalies including bicuspid aortic valve and interatrial communications. Mitral regurgitation severity varied from mild to severe. Management was individualized based on symptoms and MR severity, ranging from conservative follow-up to surgical intervention.

**Discussion:**

This series underscores the practical role of 3D TOE when TTE is equivocal: it defines leaflet morphology, distinguishes hypoplastic PML from rheumatic pathology, and detects associated anomalies that influence management. A pragmatic pathway is to suspect PML hypoplasia when MR lacks an obvious mechanism on TTE, proceed to targeted TOE/3D imaging, and tailor treatment to symptoms and MR severity.

Learning points

- Maintain a high index of suspicion for PML hypoplasia on TTE; confirmatory TOE/3D imaging is critical for characterizing leaflet morphology and coaptation abnormalities.
- Actively investigate for associated congenital anomalies (e.g. BAV, atrial septal defect or PFO), and tailor management to mitral regurgitation severity and patient symptoms.

## Introduction

Posterior mitral leaflet (PML) hypoplasia is an uncommon congenital malformation, typically recognized in childhood.^1^ Adult presentation is rare and often under-recognized, with reports ranging from asymptomatic incidental findings to severe mitral regurgitation (MR) or embolic events.^2^ Accurate diagnoses requires a high index of suspicion, as subtle features may be easily overlooked on transthoracic echocardiography (TTE). Transoesophageal echocardiography (TOE), especially with three-dimensional (3D) reconstruction, is critical for confirmation and assessment of associated anomalies. We present three adult patients with hypoplastic PML, each with distinct clinical presentations, associated conditions, and management strategies.

## Summary figure

**Figure:**
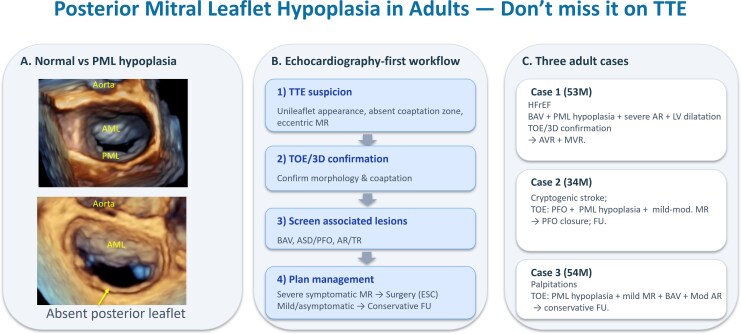
PML hypoplasia in adults is a rare and under-recognized congenital anomaly. Suspicion may arise on transthoracic echocardiography, while confirmatory TOE with three-dimensional imaging is essential. Cases often coexist with congenital anomalies such as bicuspid aortic valve or interatrial communications, and management should be tailored to mitral regurgitation severity and symptoms.

## Patient 1: PML hypoplasia with BAV

A 53-year-old man presented with persistent chest discomfort and exertional dyspnoea. His history included hypertension, persistent atrial fibrillation requiring multiple cardioversions, and chronic heart failure. A prior TTE performed at an external centre had shown a mildly dilated left ventricle (LV) with LVEF 48% (Simpson’s biplane), bicuspid aortic valve (BAV) with moderate regurgitation, moderate MR, and an aortic root diameter dilatation.

**Figure 1:**
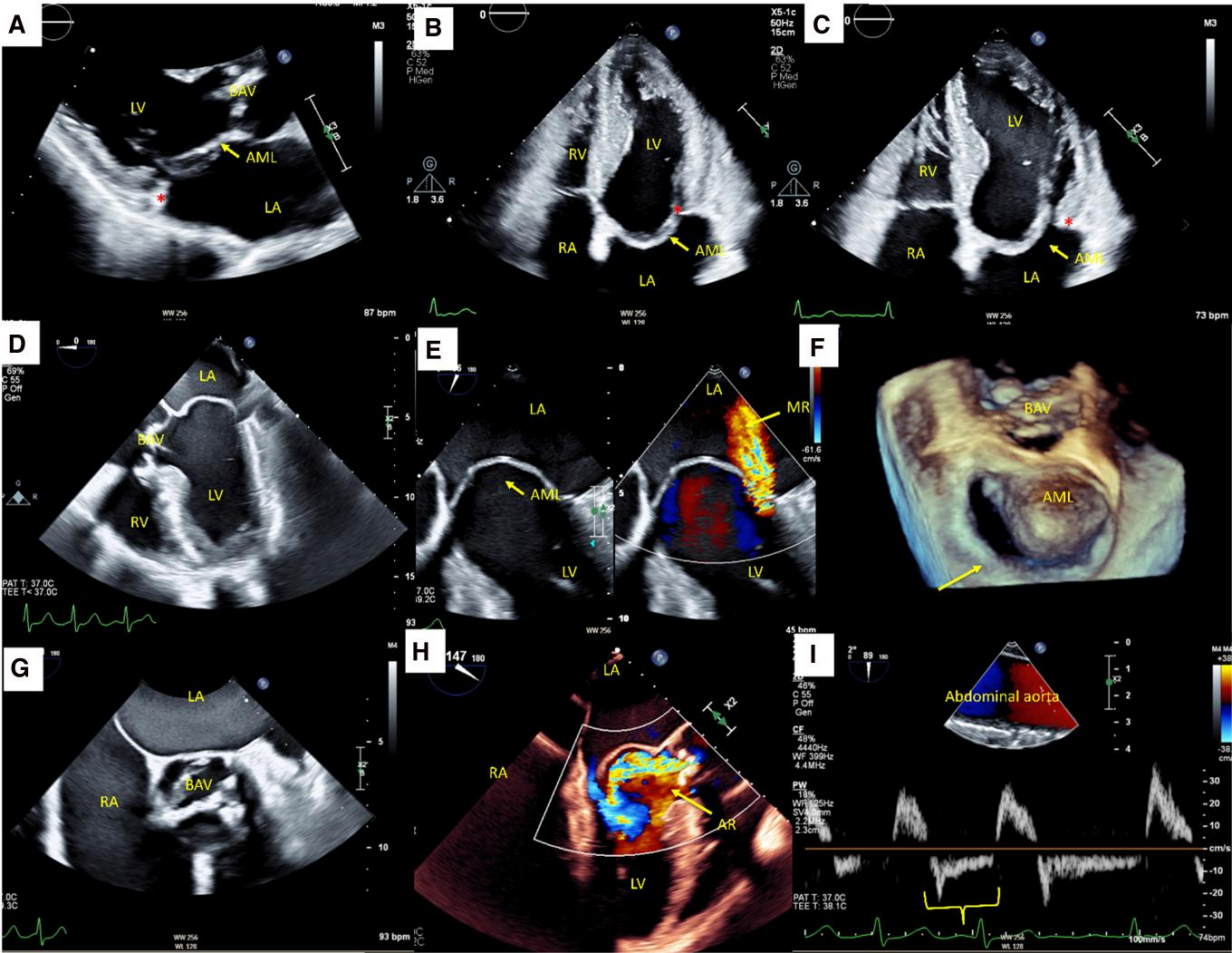
Echocardiographic assessment of PML hypoplasia with associated fusion type BAV and mixed valvular disease. (*A*) Transthoracic echocardiography (TTE), parasternal long-axis view, showing elongated anterior mitral leaflet (AML, yellow arrow), fusion type BAV, and dilated left ventricle (LV); the PML is hypoplastic (red asterisk). (*B* and *C*) Transthoracic echocardiography (TTE), apical four-chamber views in end-systole (*B*) and diastole (*C*), demonstrating an absent PML (red asterisk) and elongated AML (yellow arrows) with dilated LV and left atrium (LA). (*D*) Transoesophageal echocardiography (TOE), mid-oesophageal long-axis view at 0°, confirming BAV and hypoplastic posterior leaflet. (*E*) TOE, mid-oesophageal two-chamber view with colour Doppler, showing eccentric mitral regurgitation (MR) due to absent posterior leaflet. (*F*) TOE, three-dimensional en-face view of the mitral valve from the left atrial perspective, illustrating elongated AML and absent PML. (*G*) TOE, mid-oesophageal short-axis view, showing BAV morphology. (*H*) TOE, mid-oesophageal long-axis view with colour Doppler, demonstrating severe aortic regurgitation (AR). (*I*) TOE, abdominal aortic pulsed-wave Doppler showing holodiastolic flow reversal consistent with severe AR. BAV, bicuspid aortic valve; PML, posterior mitral leaflet; RA, right atrium; RV, right ventricle.

On admission, he was in atrial fibrillation with a systolic apical murmur. Laboratory tests and troponins were normal. Repeat TTE demonstrated a mildly dilated LV with LVEF 45%, fusion type BAV^3^ with at least moderate stenosis (peak aortic velocity 2.4 m/s, mean gradient 12 mmHg, and dimensionless index 0.45) and severe eccentric regurgitation (holodiastolic flow reversal in the descending aorta with an end-diastolic velocity > 20 cm/s), an elongated anterior mitral leaflet (AML) with moderate MR (difficult quantification due to an eccentric jet), and absent leaflet coaptation (*Figure 1A–C*). The aortic root measured 5 cm at the sinuses of Valsalva, and the left atrium was severely dilated. TOE-confirmed prolapse of the AML and a rudimentary PML consistent with congenital hypoplasia (*Figure 1D–F*). The BAV had a raphe between the right and left coronary cusps, with calcification and restricted excursion leading to at least moderate stenosis and severe regurgitation, accompanied by holodiastolic flow reversal in the abdominal aorta (*Figure 1G–I*; Supplementary material online, *Videos S1* and *S2*).

He met guideline-based criteria for aortic root/valve surgery due to severe eccentric AR with BAV and root dilatation (confirmed with CT aortography) in a symptomatic patient with LV remodelling and LVEF ∼45%–50%. Concomitant mitral surgery was planned because MR was mechanistically complex (AML prolapse with rudimentary PML causing malcoaptation), unlikely to regress, and contributed to symptoms; atrial fibrillation and marked left atrial enlargement supported bi-atrial ablation and left atrial appendage exclusion.

After optimization of heart failure therapy and diuresis, he underwent successful aortic root replacement with a 27 mm valved conduit (Konect), mitral valve replacement with a 31 mm bioprosthesis (Mitris), bi-atrial ablation, and exclusion of the left atrial appendage with a 50 mm clip. Intraoperative inspection suggested rheumatic involvement, with apparent commissural fusion, a thickened and crowded subvalvular apparatus, and a diminutive PML. However, histopathological examination demonstrated degenerative changes only, including myxoid degeneration with chondromatous and osseous metaplasia involving both the mitral and aortic valves. Importantly, there was no histological evidence of inflammatory infiltrates, fibrosis, commissural fusion, or neovascularization to support an acquired rheumatic aetiology.

## Patient 2: PML hypoplasia with PFO

A 34-year-old previously healthy man presented with sudden monocular blindness, followed by transient left-sided weakness and dysarthria. Neurological deficits resolved except for mild sensory loss. He had no cardiovascular risk factors. MRI confirmed acute right cerebellar and thalamic infarcts. Laboratory, autoimmune, and thrombophilia studies were unremarkable.

**Figure 2:**
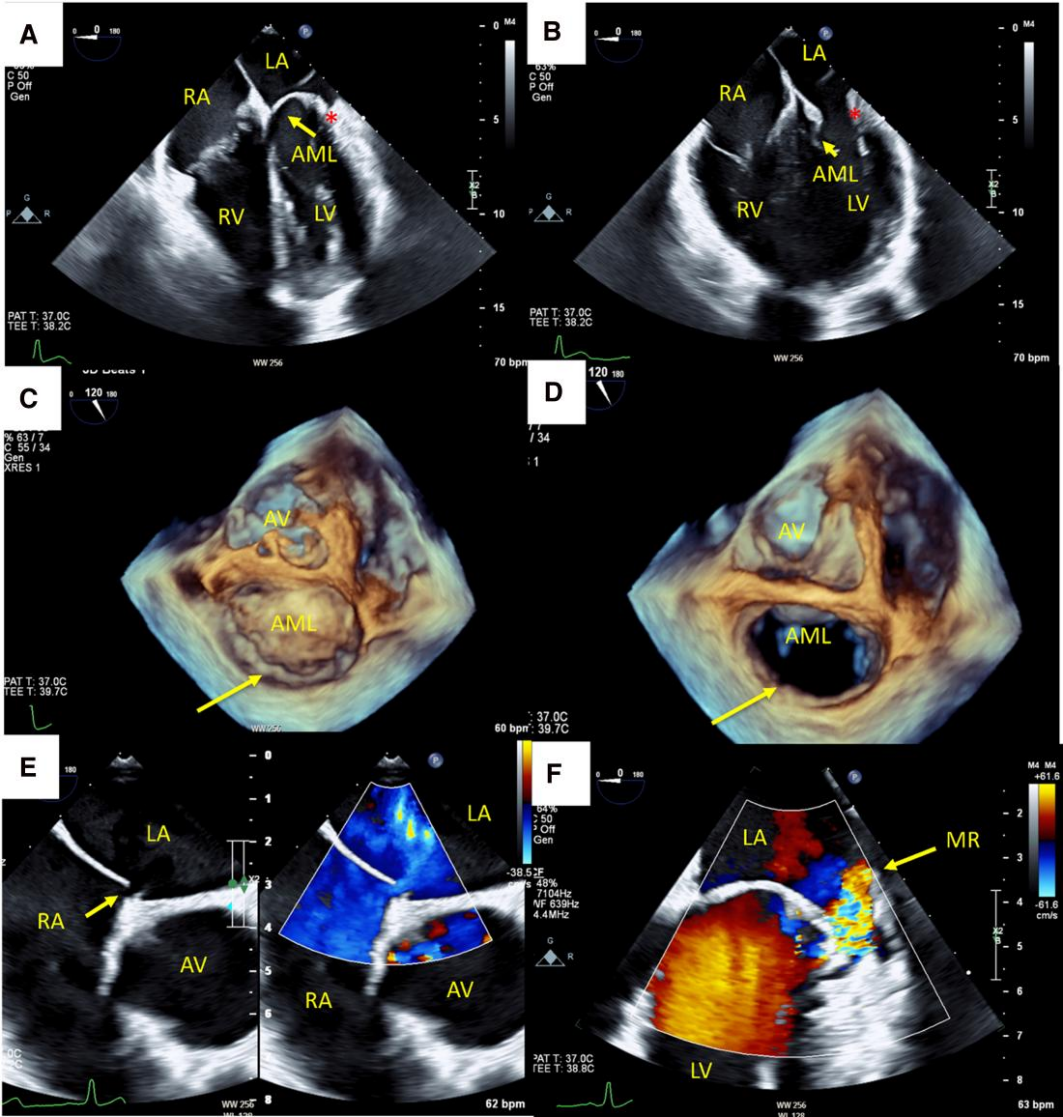
Transoesophageal echocardiographic features of PML hypoplasia with eccentric mitral regurgitation and PFO. (*A* and *B*) Transoesophageal echocardiography (TOE), mid-oesophageal four-chamber views in end-systole (*A*) and diastole (*B*), demonstrating an elongated anterior mitral leaflet (AML, yellow arrow) and a nearly absent posterior leaflet (red asterisk), consistent with hypoplasia (*C* and *D*) TOE, three-dimensional en-face views from the left atrial perspective, illustrating the elongated AML and absent posterior leaflet (yellow arrows). (*E*) TOE, mid-oesophageal short-axis view in colour compares showing the inter-auricular septum and a PFO with left-to-right shunt. (*F*) TOE, mid-oesophageal long-axis view at 0° with colour Doppler, demonstrating eccentric mild-to-moderate mitral regurgitation (MR) due to absent posterior leaflet. AV, aortic valve; AR, aortic regurgitation; LA, left atrium; LV, left ventricle; PFO, patent foramen ovale; PML, posterior mitral leaflet; RA, right atrium; RV, right ventricle.

TTE revealed a PFO, AML prolapse with myxomatous appearance, and mild-to-moderate MR in the context of a markedly hypoplastic PML, limiting accurate quantitative assessment. TOE confirmed a PFO and demonstrated an elongated anterior leaflet with a severely hypoplastic PML, resulting in eccentric mild MR (*Figure 2*; Supplementary material online, *Videos S3* and *S4*).

Given a cryptogenic ischaemic stroke in a young patient with TOE-confirmed PFO and no alternative cause identified, percutaneous PFO closure was performed without complication. Post-procedure antiplatelet therapy was prescribed per institutional protocol, with neurology follow-up.

At 6-month TTE follow-up, quantitative MR assessment using volumetric methods demonstrated a regurgitant fraction of 34% and a vena contracta width of 0.4 cm, consistent with moderate severity, PISA-derived quantification was not feasible due to the eccentric jet geometry. MR continued to be managed conservatively, with planned echocardiographic surveillance.

## Patient 3: PML hypoplasia with partial-fusion BAV

A 54-year-old man underwent evaluation for exertional palpitations. He denied chest pain or dyspnoea. Past medical history included recurrent staphylococcal skin infections and chronic sinusitis.

**Figure 3:**
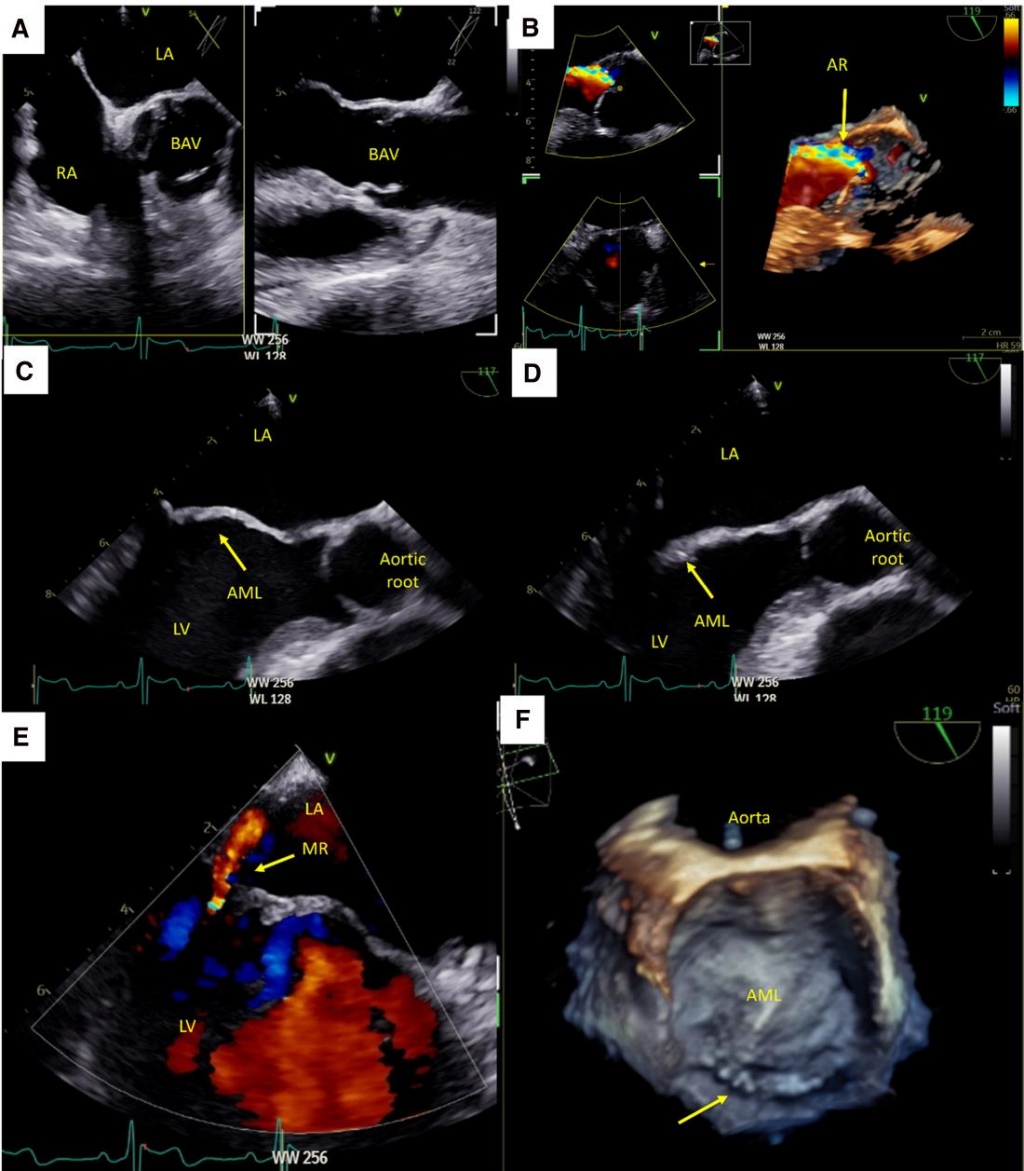
Transoesophageal echocardiographic features of PML leaflet hypoplasia with partial-fusion BAV. (*A*) Transoesophageal echocardiography (TOE), mid-oesophageal short-axis view at the level of the aortic valve (AV), demonstrating a partially fused, calcified BAV. (*B*) TOE, long-axis view with colour Doppler and 3D multi-planar reconstruction, showing an elongated anterior mitral leaflet (AML, yellow arrow) and moderate aortic regurgitation (AR). (*C* and *D*) Transoesophageal echocardiography (TOE), mid-oesophageal long-axis views in end-systole (*C*) and diastole (*D*), highlighting an elongated AML (yellow arrow), absent/rudimentary posterior leaflet, and a dilated aortic root. (*E*) TOE, long-axis view with colour Doppler, demonstrating eccentric mild mitral regurgitation (MR). (*F*) TOE, three-dimensional en-face view from the left atrial side, illustrating elongated AML and absent posterior leaflet (yellow arrow). BAV, bicuspid aortic valve; LA, left atrium; LV, left ventricle; PML, posterior mitral leaflet; RA, right atrium.

TTE demonstrated a moderately dilated LV with preserved systolic function, grade II diastolic dysfunction, an apparently tricuspid aortic valve with moderate AR, and mild MR with a unileaflet appearance. TOE confirmed a partial-fusion BAV^3^ with moderate-to-severe AR (vena contracta 0.49 cm; 3D vena contracta area 0.3 cm^2^; jet <50% LVOT; no holodiastolic reversal in descending aorta). The AML exhibited degenerative thickening, while the PML was nearly absent, consistent with hypoplasia, resulting in an eccentric mild to moderate MR (*Figure 3*; Supplementary material online, *Video S5* and *Video S6*). The patient was managed conservatively with regular clinical and echocardiographic follow-up.

## Discussion

PML hypoplasia is a rare congenital anomaly typically identified in childhood but occasionally diagnosed in adulthood, often in the context of associated conditions such as BAV and PFO.^2^ Adult cases likely represent under-recognition rather than true rarity, as diagnostic challenges arise from the subtle, easily overlooked findings on TTE.^4^ On TTE, PML hypoplasia should be suspected when the posterior leaflet appears truncated with restricted excursion and reduced coaptation length, often with an elongated anterior leaflet and a small systolic malcoaptation gap. Colour Doppler typically demonstrates an eccentric regurgitant jet with a Coanda effect, without an obvious flail or cleft. Optimized zoomed imaging and appropriate colour settings may help identify the rudimentary posterior leaflet and prompt confirmatory 3D TOE, which was crucial for definitive diagnosis and assessment of associated anomalies in all cases. Quantification of MR may nevertheless remain limited in this setting, reflecting real-world challenges in assessing eccentric MR in congenital leaflet abnormalities; furthermore, severe PML hypoplasia may limit accurate annular reconstruction, as observed in the third case. Additionally, the tertiary referral nature of our centre may have enriched the cohort with more complex presentations, introducing selection (spectrum) bias and limiting the generalizability of prevalence estimates to primary or secondary care settings. The clinical course of PML hypoplasia is heterogeneous, ranging from incidental, asymptomatic findings to severe MR with progressive left ventricular remodelling and heart failure.^4^ Surgical intervention is indicated for patients with severe symptomatic MR, according to ESC/EACTS guidelines, whereas conservative management is generally appropriate for asymptomatic patients or those with only mild MR. Importantly, in patients with moderate MR who require major cardiac surgery for another indication, such as aortic valve replacement or coronary artery bypass grafting, concomitant mitral surgery should be considered to prevent progression of regurgitation and reduce the risk of reoperation.^5^

PML hypoplasia often coexists with other congenital lesions, which must be actively sought during echocardiographic assessment.^2,4,6^ Additionally, distinguishing hypoplastic PML from rheumatic mitral valve disease is essential, as thickened and restricted leaflets may mimic end-stage rheumatic appearances, leading to diagnostic uncertainty.^5^ In such cases, histopathology and rheumatological markers can help exclude chronic rheumatic involvement.

The rarity of this entity in adulthood is supported by limited reports in the literature.^2,4^  *Table 1* summarises the patients in our case series and representative reports from the literature, illustrating the spectrum of clinical presentation, associated anomalies, and management strategies in adults with PML hypoplasia. The full list of published non-infant cases is provided in Supplementary material online, *Table S1*.

**Table 1:** Clinical characteristics, echocardiographic findings, and outcomes of posterior mitral leaflet hypoplasia in adults (index cases and selected literature)

Case	Age/sex	Presentation	3D TOE	MR severity	Associated anomalies	Management/outcome
1 (this series)	53M	Dyspnoea, chest discomfort	Yes	Mild	BAV, moderate AS, severe AR	Surgical AVR + MVR
2 (this series)	34M	Cryptogenic stroke	Yes	Mild–moderate	PFO	PFO closure; follow-up
3 (this series)	54M	Palpitations	Yes	Mild	AR	Follow-up
Bär *et al*.^1^	62F	Asymptomatic	No	Mild–moderate	None	Follow-up
de Agustin *et al*.^7^	73F	Dyspnoea	Yes	Severe	None	MVR
Parato and Masia^8^	35M	Palpitations	Yes	Severe	BAV, severe AR, non-compaction	Surgical AVR + MVR
Antit *et al*.^9^	30M	Chest pain, complete AV block	No	None	None	Dual-chamber PPM; follow-up
Bertolín-Boronat *et al*.^10^	65F	Dyspnoea, pulmonary oedema	Yes	Severe	Sinus venosus ASD, severe TR	MVR + TV annuloplasty + ASD closure

MR, mitral regurgitation; 3D TOE, three-dimensional transoesophageal echocardiography; BAV, bicuspid aortic valve; AS, aortic stenosis; AR, aortic regurgitation; PFO, patent foramen ovale; ASD, atrial septal defect; TR, tricuspid regurgitation; AVR, aortic valve replacement; MVR, mitral valve replacement; PPM, permanent pacemaker.

In summary, this case series highlights that PML hypoplasia in adults is under-recognized, often associated with other congenital anomalies, and should be actively considered when MR is present without an obvious mechanism on TTE. A systematic echocardiographic evaluation with a high index of suspicion is essential to avoid missed diagnoses and to guide appropriate management.

## Lead author biography


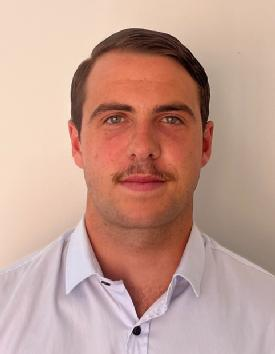


Dylan Young is a resident medical officer at Gold Coast University Hospital, Queensland, Australia. He has a strong clinical interest in cardiology, with a focus on advancing patient care through research and evidence-based practice.

## Supplementary Material

ytag160_Supplementary_Data

## Data Availability

All relevant data supporting the findings of this study are included in the article.
