# Supplementary material for: Posterior mitral leaflet hypoplasia in adults: diagnostic value of three-dimensional transoesophageal echocardiography: a case series
Source: Eur Heart J Case Rep. 2026 Apr 24;10(4):ytag160. doi: 10.1093/ehjcr/ytag160 (PMC13107961; doi:10.1093/ehjcr/ytag160)
Supplement: ytag160_Supplementary_Data [file ytag160_supplementary_data.zip › PMV Supplementary Data.docx]

# SUPPLEMENTARY DATA

**Video S1**. **PML hypoplasia with BAV. TOE mid-oesophageal views of the aortic and mitral valves**. Mid-oesophageal short-axis TOE view showing a BAV with fusion of the right and left coronary cusps, severe cusp calcification, and severe regurgitation. Right panel: TOE mid-oesophageal long-axis view demonstrating an elongated anterior mitral leaflet with absent posterior leaflet.

**Video S2. PML hypoplasia with BAV.3D TOE of the mitral valve.** En face 3D TOE view of the mitral valve showing a prominently elongated anterior mitral leaflet and complete absence of the posterior leaflet.

**Video S3. PML hypoplasia with PFO. TOE long-axis with colour Doppler and 2D imaging**. Left panel: TOE mid-oesophageal long-axis view with and without colour Doppler, demonstrating a patent foramen ovale (arrow) with left-to-right shunting. Right panel: TOE long-axis view at 120° showing an elongated anterior mitral leaflet with absent posterior leaflet.

**Video S4. PML hypoplasia with PFO. 3D TOE of the mitral valve.** En face 3D TOE view of the mitral valve showing a prominently elongated anterior mitral leaflet and complete absence of the posterior leaflet.

**Video S5. PML hypoplasia with partial-fusion BAV. 3D TOE of the aortic and mitral valves.** Left panel: 3D TOE view of the aortic valve demonstrating partial-fusion BAV with associated aortic regurgitation. Right panel: TOE long-axis view at 120° showing an elongated anterior mitral leaflet with absent posterior leaflet.

**Video S6. PML hypoplasia with partial-fusion BAV. 3D TOE of the mitral valve.** 3D TOE view of the mitral valve showing a prominently elongated anterior leaflet and absent

posterior leaflet, illustrating the challenge of accurate definition even with advanced 3D imaging.

**Table S1.** Clinical characteristics, echocardiographic findings, and outcomes of reported non- infant cases of posterior mitral leaflet hypoplasia (including present series).

|  | Age and Gender | Presenting complaint | 3D Echo | Mitral valve regurgitation | Associated disease/anomaly | Management/Outcome |
| --- | --- | --- | --- | --- | --- | --- |
| **1.** | **53-year-old male** | **Chest discomfort, exertional dyspnoea** | **Yes** | **Moderate MR** | **BAV, mild AS, severe AR** | **Surgical MVR and AVR** |
| **2.** | **34-year-old male** | **Left eye weakness, left**  **hemiparesis (stroke)** | **Yes** | **Mild-Moderate MR** | **PFO** | **PFO closure, regular follow-up** |
| **3.** | **54-year-old male** | **Palpitations on exertion** | **Yes** | **Mild MR** | **BAV and moderate AR** | **Regular follow-up** |
| 4. | 62-year-old female | Asymptomatic | No | Mild-to-moderate MR | Nil | Regular follow-up |
| 5. | 62-year-old male | Asymptomatic | No | No MR | Previous AVR (for severe AR) | Regular follow-up |
| 6. | 72-year-old  female | Asymptomatic | No | Mild MR | Moderate AR. | Regular follow-up |
| 7. | 18-year-old female | Asymptomatic | No | Mild MR | Nil | Regular follow-up |
| 8. | 17-year-old female | Asymptomatic | No | Mild MR | Nil | Regular follow-up |
| 9. | 46-year-old female | Asymptomatic | No | Mild MR | Nil | Regular follow-up |
| 10. | 54-year-old  female | Asymptomatic | No | Mild MR | Secundum-type  ASD | Regular follow-up |
| 11. | 62-year-old male | Dyspnoea | No | Severe MR | Nil | Surgical MVR |

| 12. | 73-year-old female | Dyspnoea | Yes | Severe MR | Nil | Surgical MVR |
| --- | --- | --- | --- | --- | --- | --- |
| 13. | 45-year-old female | Dyspnoea on exertion | Yes | Mild MR | Obstructive SAM | Regular follow-up |
| 14. | 51-year-old male | Dyspnoea, acute pulmonary oedema | Yes | Severe MR | BAV, severe AR | Surgical mitral valve repair, AVR |
| 15. | 76-year-old male | Dyspnoea, acute pulmonary oedema | Yes | Severe MR | Nil | Surgical mitral valve repair |
| 16. | 66-year-old female | Dyspnoea | No | Severe MR | Nil | Surgical MVR |
| 17. | 22-year-old male | Recurrent  palpitations | No | No MR | Nil | Regular follow-up |
| 18. | 24-year-old male | Atypical chest pain | Yes | Mild MR | Nil | Regular follow-up |
| 19. | 69-year-old female | Dyspnoea on exertion | Yes | Severe MR | Nil | Surgical MVR |
| 20. | 35-year-old male | Palpitations | Yes | Severe MR | BAV, severe AR, non-  compaction cardiomyopathy | Surgical MVR and AVR |
| 21. | 21-year-old male | Asymptomatic | No | Mild MR | Nil | Regular follow-up |
| 22. | 65-year-old female | Dyspnoea on exertion | Yes | Severe MR | Nil | Surgical mitral valve repair |
| 23. | 59-year-old male | Asymptomatic. | No | Mild MR | Nil | Regular follow-up |
| 24. | 48-year-old male | Dyspnoea on exertion | Yes | Severe MR | BAV, PFO,  non-compaction cardiomyopathy.  Previous ductus arteriosus repair. | Surgical MVR and AVR. Subsequent PPM insertion. |
| 25. | 26-year-old male | Pleuritic chest pain and dyspnoea | No | No MR | Nil | Regular follow-up |
| 26. | 72-year-old male | Dyspnoea | Yes | Severe MR | Severe TR. Previous BAV  with AVR. | Regular follow-up. (Declined surgical  intervention). |
| 27. | 30-year-old male | Chest pain, dyspnoea | No | No MR | Nil on imaging. ECG showed complete  atrioventricular block. | Dual-chamber PPM. Regular follow-up. |
| 28. | 60-year-old male | Asymptomatic | Yes | Severe MR | BAV, moderate AR | Surgical MVR and AVR. Subsequent  PPM insertion. |
| 29. | 45-year-old- male | Palpitations and dyspnoea | Yes | Severe MR | Secundum type ASD | Regular follow-up.  (refused surgical intervention). |
| 30. | 59-year-old female | Dyspnoea | Yes | Severe MR | Secundum-type ASD | Surgical MVR and ASD closure |
| 31. | 65-year-old- female | Dyspnoea, acute pulmonary oedema | Yes | Severe MR | Inferior sinus venosus ASD, severe TR.  History of surgical ostium  secundum ASD repair. | Surgical mitral and tricuspid annuloplasty. Sinus venosus ASD closure. |
| 32. | 44-year-old male | Acute respiratory distress syndrome | Yes | Severe MR | BAV, moderate AR | Surgical MVR and AVR |
| 33. | 76-year-old female | Dyspnoea, palpitations | Yes | Severe MR | Nil | Surgical MVR |
| 34. | 82-year-old male | Dyspnoea, fatigue | Yes | Severe MR | Previous TAVR for severe AS | Regular follow-up. (not surgical candidate) |

| 35. | Retrospective cohort study  44 Cases  -22 male  -22 female  Median age 31  (ages 23-44.5) | 22 cases reported dyspnoea | All patients received TTE and TOE  TOE with three dimensional multiplanar reconstruction in 30 of 44 patients | 14 Moderate MR  11 Severe MR | 13 BAV  2 unicuspid unicommissural aortic valve  7 Secundum ASD  1 Primum ASD | 7 MVR  14 MVR + other surgery  23 Regular follow- up |
| --- | --- | --- | --- | --- | --- | --- |

Abbreviations: MR = mitral regurgitation; MVR = mitral valve replacement; AVR = aortic valve replacement; AS = aortic stenosis; AR = aortic regurgitation; BAV = bicuspid aortic valve; PFO = patent foramen ovale; ASD = atrial septal defect; SAM = systolic anterior motion; TR = tricuspid regurgitation; PPM = permanent pacemaker; TAVR = transcatheter aortic valve replacement; TTE = transthoracic echocardiography; TOE = transoesophageal echocardiography.

# Posterior Mitral Leaflet Hypoplasia in Adults: Diagnostic Value of Three-Dimensional Transoesophageal Echocardiography - A Case Series

**Table S1 references:**

1-3. Posterior Mitral Leaflet Hypoplasia in Adults: Diagnostic Value of Three-Dimensional Transoesophageal Echocardiography – A Case Series (this paper)

4–6. Bär H, Siegmund A, Wolf D, Hardt S, Katus HA, Mereles D. Prevalence of asymptomatic mitral valve malformations. *Clin Res Cardiol* 2009;98:305–309. DOI: 10.1007/s00392-009-0734-6

7–9. Kanagala P, Baker S, Green L, Houghton AR. Functionally uni-leaflet mitral valves in a family: A case series. *Eur Heart J Cardiovasc Imaging* 2010;11:E27. DOI: 10.1093/ehjci/jeq125

1. Heper G, Yetkin E, Senen K. Absence of posterior mitral leaflet with secundum atrial septal defect. *Ann Thorac Surg* 2010;90:2055–2057. DOI: 10.1016/j.athoracsur.2010.07.014
2. Ozkan H, Tiryakioglu O, Cetinkaya AS, Uyanik EC, Bozat T. Agenesis of the mitral posterior leaflet in elderly. *Ann Thorac Surg* 2014;97:319–321. DOI: 10.1016/j.athoracsur.2013.04.134
3. de Agustin JA, de Diego JJG, Garcia-Fernandez MA, Rodrigo JL, Marcos-Alberca P, Almeria C, et al. Severe hypoplasia of the posterior mitral leaflet: A rare cause of congenital mitral regurgitation assessed by three-dimensional transesophageal echocardiography. *Int J Cardiol* 2014;177:e131–e132. DOI: 10.1016/j.ijcard.2014.09.019
4. Bezgin T. Mitral valve with a single leaflet. *Turk Kardiyol Dern Ars* 2014;42:80–82.

DOI: 10.5543/tkda.2014.44380

1. Saura D, Oliva MJ, Sanchez-Galian MJ, Gonzalez J, Caballero L, Mateo-Martinez A, et al. Real-time three-dimensional transesophageal echocardiographic evaluation of the association of bicuspid aortic valve and mitral posterior leaflet hypoplasia. *Int J Cardiol* 2015;195:334–335. DOI: 10.1016/j.ijcard.2015.04.214
2. Yazdan-Ashoori P, Rohani A, Mulji AS, Van Spall HGC. Hypoplasia of the posterior mitral valve leaflet detected in late adulthood. *Eur Heart J* 2015;36:456. DOI: 10.1093/eurheartj/ehu513
3. Joshi V, Laurie K, Skoyles J, Richens D. Severe mitral regurgitation secondary to atresia of the posterior mitral valve leaflet in the adult: Is repair always best practice? *Thorac Cardiovasc Surg Rep* 2014;4:034–036. DOI: 10.1055/s-0034-1394104
4. Shah J, Jain T, Shah S, Mawri S, Ananthasubramaniam K. Rare case of unileaflet mitral valve. *J Cardiovasc Ultrasound* 2016;24:168. DOI: 10.4250/jcu.2016.24.2.168
5. Fazlinezhad A, Alvandi Azari M, Bigdellu L. Severe hypoplasia of posterior mitral valve leaflet presented with atypical chest pain: A case report. *Razavi Int J Med* 2016;5:e41501. DOI: 10.5812/rijm.41501
6. Bacich D, Braggion G, Faggian G. Hypoplasia of the posterior mitral leaflet: A rare cause of mitral regurgitation in adulthood. *Echocardiography* 2017;34:949–950. DOI: 10.1111/echo.13556

20–21. Parato V, Masia S. Hypoplasia or absence of posterior leaflet: A rare congenital anomaly of the mitral valve in adulthood—case series. *J Cardiovasc Echogr* 2018;28:45. DOI: 10.4103/jcecho.jcecho_6_18

22. Arasaratnam K, Tomlinson S, Dahiya A, Lo A, Jalali H, Prasad SB. Surgical repair of a unileaflet mitral valve: A rare congenital abnormality and a novel surgical approach. *CASE (Phila)* 2020;4:420–428. DOI: 10.1016/j.case.2020.07.005

23–24. Kadlečková A, Ioniţa OR, Weichet J, Kačer P, Línková H. Hypoplasia of the posterior mitral leaflet—are we familiar with it? *Cor Vasa* 2021;63:502–505. DOI: 10.1016/j.corvasa.2021.07.004

1. Grandez C, Skenderi S. Unileaflet mitral valve in patient with marfanoid habitus. *CASE (Phila)* 2023;7:10–13. DOI: 10.1016/j.case.2023.02.001
2. Emami E, Shemshadi S, Farrashi M. Aplastic posterior mitral leaflet with an unusual clinical course. *Res Cardiovasc Med* 2024;13:134–136. DOI: 10.34172/rcm.2024.134
3. Antit S, Soumer K, Abdelhedi M, Zidi O, Zakhama L. Incidental findings of congenital unileaflet mitral valves in young patient presenting complete atrioventricular block. *Pediatr Cardiol* 2025;46:1003–1007. DOI: 10.1007/s00246-024-03602-w
4. Velarde-Acosta K, Moscoso Ramirez JY, Medina-Maguiña JM, Baltodano-Arellano R. Hypoplastic posterior leaflet mitral valve associated with bicuspid aortic valve. *CASE (Phila)* 2024;8(3):151–156. DOI: 10.1016/j.case.2023.12.025
5. Pourafkari N, Baghbani-Oskouei A, Toufan M, Ghaffari S, Nader ND. Hypoplastic posterior mitral valve leaflet: a case report and review of the literature. *Echocardiography* 2018;35(7):1052–1055. DOI: 10.1111/ECHO.13898
6. Gabaldón-Pérez A, Marcos-Garcés V, Muñoz Gil J, Chorro-Gascó FJ, Santas E. Hypoplasia of the posterior mitral valve leaflet: Don’t forget to look beyond the mitral valve. *Echocardiography* 2020;38(1):142–143. DOI: 10.1111/echo.14910
7. Bertolín-Boronat C, de la Espriella R, Monmeneu JV, López-Lereu MP, Santas E, Merenciano-González H, et al. A rare case of inferior sinus venosus atrial septal defect and concomitant hypoplasia of the posterior mitral valve leaflet. *Echocardiography* 2024;41(9):e15923. DOI: 10.1111/echo.15923.

32-34. Zavadil C, Neuhauser T, Eichhorn F, Cesnjevar R, Dittrich S, Haas NA. Posterior Mitral Valve Hypoplasia: Three Clinical Cases and a Review of the Literature. *Biomedicines* 2025;13(5):1078. DOI: 10.3390/biomedicines13051078.

35. Karaduman A, Yılmaz C, Balaban İ, Aytürk M, Sarı M, Bayram Z, et al. An echocardiographic study of a rare cause of mitral regurgitation: Hypoplastic posterior mitral valve leaflet. *Anatol J Cardiol* 2025;29:124–131. DOI: 10.14744/AnatolJCardiol.2024.50302
